# The Association of an Alpha-2 Adrenergic Receptor Agonist and Mortality in Patients With COVID-19

**DOI:** 10.3389/fmed.2021.797647

**Published:** 2022-01-04

**Authors:** John L. Hamilton, Mona Vashi, Ekta B. Kishen, Louis F. Fogg, Markus A. Wimmer, Robert A. Balk

**Affiliations:** ^1^Department of Orthopedic Surgery, Rush University Medical Center, Chicago, IL, United States; ^2^Division of Pulmonary and Critical Care Medicine, Department of Internal Medicine, Rush University Medical Center, Chicago, IL, United States; ^3^Bioinformatics and Biostatistics Core, Rush University Medical Center, Chicago, IL, United States; ^4^Department of Community, Systems and Mental Health Nursing, Rush University Medical Center, Chicago, IL, United States

**Keywords:** coronavirus disease 2019 (COVID-19), dexmedetomidine, mortality, severe acute respiratory syndrome coronavirus 2 (SARS-CoV-2), alpha-2 adrenergic receptor agonist

## Abstract

There is a need for treatments to reduce coronavirus disease 2019 (COVID-19) mortality. Alpha-2 adrenergic receptor (α_2_ AR) agonists can dampen immune cell and inflammatory responses as well as improve oxygenation through physiologic respiratory parameters. Therefore, α_2_ AR agonists may be effective in reducing mortality related to hyperinflammation and acute respiratory failure in COVID-19. Dexmedetomidine (DEX) is an α_2_ AR agonist used for sedation. We performed a retrospective analysis of adults at Rush University System for Health hospitals between March 1, 2020 and July 30, 2020 with COVID-19 requiring invasive mechanical ventilation and sedation (*n* = 214). We evaluated the association of DEX use and 28-day mortality from time of intubation. Overall, 28-day mortality in the cohort receiving DEX was 27.0% as compared to 64.5% in the cohort that did not receive DEX (relative risk reduction 58.2%; 95% CI 42.4–69.6). Use of DEX was associated with reduced 28-day mortality on multivariable Cox regression analysis (aHR 0.19; 95% CI 0.10–0.33; *p* < 0.001). Adjusting for time-varying exposure to DEX also demonstrated that DEX was associated with reduced 28-day mortality (aHR 0.51; 95% CI 0.28–0.95; *p* = 0.03). Earlier DEX use, initiated <3.4 days from intubation, was associated with reduced 28-day mortality (aHR 0.25; 95% CI 0.13–0.50; *p* < 0.001) while later DEX use was not (aHR 0.64; 95% CI 0.27–1.50; *p* = 0.30). These results suggest an α_2_ AR agonist might reduce mortality in patients with COVID-19. Randomized controlled trials are needed to confirm this observation.

## Introduction

The coronavirus disease 2019 (COVID-19) caused by severe acute respiratory syndrome coronavirus 2 (SARS-CoV-2) has caused over 5.3 million deaths worldwide to date (1). Severe complications of COVID-19 include acute respiratory failure and multi-organ dysfunction. Causes of severe COVID-19 complications and death include a hyperimmune response precipitating cytokine storm and hypoxemia caused by pulmonary dysfunction (2). An initial trigger of this hyperimmune response and pulmonary dysfunction is infection of lung alveolar cells, vascular endothelial cells, as well as other cell types with SARS-CoV-2 with further viral replication within cells and subsequent cell death (3–5). Dead cell debris and viral components bind to pattern recognition receptors (PRRs) of innate immune cells, triggering immune cell recruitment and activity. These activated immune cells can subsequently cause damage to host tissue through mechanisms such as release of reactive oxygen species (ROS) or cytokine production and release, causing further immune cell activation as well as organ and vascular dysfunction. With absence of clearance of infection, a perpetual dysregulated immune response can occur resulting in cytokine storm (3, 4).

Severe COVID-19 complications are closely linked to a hyperinflammatory state. For this reason, treatments that suppress the immune system and inflammation such as but not limited to corticosteroids, interleukin-6 (IL-6) inhibitors, and Janus kinase (JAK) inhibitors have been studied as treatments to improve COVID-19 outcomes (6–8). The strongest current evidence demonstrate corticosteroids reduce COVID-19 mortality (9, 10). Corticosteroids have broad immunosuppressive effects on both the innate and adaptive immune response. While the immunosuppressive benefits of corticosteroids have proven a mortality benefit to patients with moderate to severe COVID-19 illness (9, 10), there are concerns that such a broad immunosuppressant could delay viral clearance. Corticosteroids have delayed viral clearance in patients with novel coronavirus infections such as severe acute respiratory syndrome (SARS) and Middle East respiratory syndrome (MERS), leading to concerns of delayed viral clearance in COVID-19 (11–13); there have been heterogenous reports as to whether this is the case for COVID-19 (14, 15). A limitation of corticosteroids is that their use provides no benefit and potential harm to patients with less severe COVID-19 not requiring supplemental oxygen (9), which may be due to the broad immunosuppressive actions of corticosteroids as well as other associated side effects.

Overall, corticosteroids, while beneficial at the right time and dose, may be a double-edged sword in COVID-19, and alternative or adjunct immunomodulatory agents may be of value for treating patients with COVID-19. A relatively new development in the understanding of the immune response and inflammation is the role that catecholamines and catecholamine receptors, such as the alpha-1 adrenergic receptor (α_1_ AR) and alpha-2 adrenergic receptor (α_2_ AR), play in immune cell activity and inflammatory cytokine production. Staedtke et al. (16) demonstrated that either suppression of catecholamine (norepinephrine and epinephrine) synthesis or suppression of catecholamine signaling with an α_1_ AR antagonist reduced inflammatory cytokine production and inflammatory injury and improved survival in mouse models of cytokine storm. These findings were hypothesized to be applicable to COVID-19 treatment (17), and, indeed, retrospective cohort analysis demonstrated that use of an α_1_ AR antagonist was associated with up to a 74% relative risk reduction for death in patients hospitalized with COVID-19 (18). A clinical trial is underway to further investigate these findings: ClinicalTrials.gov Identifier: NCT04365257.

As opposed to catecholamine signaling at the α_1_ AR receptor, stimulation of the α_2_ AR receptor serves as a negative feedback regulator of catecholamine release, subsequently decreasing catecholamine mediated signaling (19–23). Therefore, an α_2_ AR agonist may function similarly to an α_1_ AR antagonist by suppressing catecholamine signaling. The potential of suppression of the sympathetic nervous system, catecholamine signaling, or specifically α_2_ AR agonism to reduce COVID-19 mortality has been discussed (24–26).

Aside from suppression of catecholamine release, α_2_ AR agonists have other potential direct immunomodulatory effects such as maintaining endothelial junction integrity and attenuating microcirculatory derangements, as well as reducing immune cell recruitment and activity at the site of an inflammatory stimulus (27–30); our own preliminary animal data support these findings and demonstrate a substantially diminished localization of immune cell activity to a local inflammatory stimulus in response to an α_2_ AR agonist (unpublished). Furthermore, α_2_ AR agonists have been reported to suppress inflammatory cytokine production and provide organ protection (blood vessels, heart, brain, kidney) through anti-inflammatory and sympatholytic activities (28, 29). An α_2_ AR agonist (clonidine) reduced lung edema and improved survival in a murine viral (influenza A) lethal infection model (31). Aside from immunomodulatory properties of α_2_ AR agonists, other reported benefits of α_2_ AR agonists include reduced agitation and improved ventilator compliance, improved respiratory mechanics, as well as enhanced hypoxic pulmonary vasoconstriction and improvement in ventilation / perfusion ratio (25, 26, 32–35). Others have reviewed the mechanisms of and hypothesized on the potential therapeutic benefit of using an α_2_ AR agonist, such as dexmedetomidine (DEX) or clonidine, to mitigate COVID-19 morbidity and mortality (24–26). Clinical trials are currently underway investigating α_2_ AR agonist use in COVID-19 outcomes and immunomodulation: ClinicalTrials.gov Identifiers: NCT04413864 and NCT04358627.

There are currently approved clinical indications for use of α_2_ AR agonists, such as clonidine (e.g., hypertension), tizanidine (spasticity), and DEX (e.g., sedation). At Rush University System for Health (RUSH) hospitals, DEX is often used as a sedative for patients in the ICU receiving invasive mechanical ventilation, including in critically ill patients with COVID-19. Due to this specific usage of the α_2_ AR agonist DEX, we investigated the association of DEX use and mortality outcomes in critically ill patients with COVID-19 on retrospective analysis. Because DEX is predominately employed as an ICU sedative, and has an FDA approved indication for use in intubated and mechanically ventilated patients (36), we further restricted our patient population to patients receiving sedation for invasive mechanical ventilation.

## Materials and Methods

### Data Sources

Data was collected from electronic medical records (EMRs) of RUSH hospitals: Rush University Medical Center; Rush Copley Medical Center; and Rush Oak Park Hospital. Deidentified data was collected from the EMRs by the Rush Bioinformatics and Biostatistics Core. This study received expedited approval by the Institutional Review Board at Rush University Medical Center. All authors analyzed the data.

### Study Population

We identified patients admitted at RUSH hospitals from March 1, 2020 to July 30, 2020. Patients were included if they were of adult age (≥18), had a diagnosis of COVID-19, had acute respiratory distress syndrome or related diagnosis, and received intubation and sedation. We excluded patients that had a diagnosis of autoimmune disease or if tocilizumab was administered during hospital admission; these patients were excluded because a number of patients with COVID-19 were trialed early in the pandemic with tocilizumab, and both autoimmune disease or associated prescribed medications or tocilizumab can alter the immune system and immune response. Corticosteroids were not excluded because within the majority of time of the study period, there were no standard practices or guidelines for using this medication in the study population at RUSH hospitals. Early in the COVID-19 pandemic, there was no clear indication to provide early corticosteroid treatment at the time of oxygen support or invasive mechanical ventilation to reduce mortality. Our analysis was conducted between March 1, 2020 and July 30, 2020. Based on emerging randomized controlled trials (RCTs) reporting a mortality benefit of corticosteroid use (9, 10), it was only from July 2020 onward that early corticosteroid treatment became standard practice at RUSH hospitals. Patients meeting criteria were separated based on use of α_2_ AR agonist dexmedetomidine (DEX group) or patients that did not receive dexmedetomidine (No DEX group).

### Study End Points

We assessed 28-day mortality between the DEX and No DEX groups from the start time of intubation. Our primary tool to assess 28-day mortality was multivariable Cox proportional hazards regression. Within this Cox regression model, DEX and covariates chosen a priori based on greatest potential influence on mortality were included. Covariates besides DEX included the following: (i) age at hospital admission; (ii) body mass index (BMI) at hospital admission; (iii) modified Charlson Comorbidity Index (mCCI) at hospital admission; (iv) partial pressure of arterial oxygen to the fraction of inspired oxygen (Pao_2_/Fio_2_) at intubation; (v) modified sequential organ failure assessment (mSOFA) at intubation; vi) corticosteroid use; vii) prone positioning use. The mCCI was calculated as described by Quan et al. (37). The Pao_2_/Fio_2_ and mSOFA were calculated as the worst value over 24 h from time of intubation (38). In the mSOFA score calculation, the nervous system SOFA component score was removed, since patients in this study were assessed while under sedation. In addition, 28-day mortality between the DEX and No DEX groups using multivariable Cox proportional hazards regression accounting for time varying exposure to the drug under investigation (DEX) from time of intubation adjusting for immortal time bias was performed as previously described (12); covariates addressed above, chosen a priori for potential influence on mortality, were also included.

### Statistical Analysis

For continuous variables, independent samples *t*-tests were performed. For each continuous variable, a Levene's test for equality of variances was performed. With significance on Levene's test for heterogeneity of variances, a Mann-Whitney U test was performed. Continuous variable data are displayed as mean ± 95% confidence interval (CI). Categorical variables were assessed with a Pearson's chi-squared test. If any expected count in a 2 x 2 table was <5, a Fisher's exact test was performed. Categorical variables are displayed as counts and calculated as percentage within the group. We used *p* < 0.05 as the threshold for significance.

Mortality outcomes for DEX use and other covariates were evaluated with adjusted hazard ratios (aHR) with respective 95% CI and *p* < 0.05 for significance. Simple imputation using the mean of the immediate preceding and succeeding most severe value over 24 h was used for missing values for Pao_2_/Fio_2_ and mSOFA scores within the 24 h time period of interest (intubation) (38–40). A complete case analysis was performed (41). All analyses were performed using SPSS version 27.

## Results

### Baseline Characteristics

From March 1, 2020 to July 30, 2020, a total of 214 patients met criteria for the analysis. A total of 152 patients were in the DEX group. The remaining patients (*n* = 62) were in the No DEX group. Patient demographic characteristics and comorbidities at hospital admission were evaluated between groups (Table 1). Age was similar between the DEX and No DEX group (60.1 vs. 59.1 years; *p* = 0.83). Gender, race, and ethnicity were similar between groups. Within the DEX group, there was a higher proportion of patients with hypertension (78.9 vs. 61.3%; *p* = 0.008) and coronary artery disease (23.0 vs. 11.3%; *p* = 0.05). Active cancer and types of chronic respiratory disease, immunosuppression, kidney disease, liver disease, and metabolic disease were similar between groups. BMI was similar in the DEX group and No DEX group (33.5 vs. 34.7 kg/m^2^; *p* = 0.36). The mCCI trended higher (worse) but was not statistically significant in the DEX group vs. No DEX group (2.5 vs. 1.8; *p* = 0.07) (Table 1).

**Table 1:** Patient baseline characteristics at hospital admission.

**Characteristics**	**Dexmedetomidine** ***n* = 152**	**No Dexmedetomidine** ***n* = 62**	***p-value***
Age	60.1 (58.1–62.2)	59.1 (54.9–63.2)	0.83
Male sex	95 (62.5%)	38 (61.3%)	0.87
Race			
American Indian or Alaska Native	0 (0.00%)	0 (0.00%)	>0.999
Asian	5 (3.3%)	1 (1.6%)	0.67
Black or African American	52 (34.2%)	27 (43.5%)	0.20
White	38 (25.0%)	15 (24.2%)	0.90
Other / Not specified	57 (37.5%)	19 (30.6%)	0.34
Ethnicity			
Hispanic or Latino	66 (43.4%)	20 (32.3%)	0.13
Not Hispanic or Latino	84 (55.3%)	40 (64.5%)	0.21
Other / Not specified	2 (1.3%)	2 (3.2%)	0.58
Active Cancer	13 (8.6%)	5 (8.1%)	0.91
Cardiovascular disease			
Hypertension	120 (78.9%)	38 (61.3%)	**0.008**
Coronary artery disease	35 (23.0%)	7 (11.3%)	**0.05**
Congestive heart failure	38 (25.0%)	14 (22.6%)	0.71
Chronic respiratory disease			
Asthma	13 (8.6%)	7 (11.3%)	0.53
COPD	25 (16.4%)	10 (16.1%)	0.95
Interstitial pulmonary disease	7 (4.6%)	1 (1.6%)	0.44
Obstructive sleep apnea	21 (13.8%)	10 (16.1%)	0.66
Immunosuppression			
HIV	1 (0.66%)	0 (0.00%)	>0.999
History of organ transplant	3 (2.0%)	3 (4.8%)	0.36
Kidney disease			
Chronic	49 (32.2%)	13 (21.0%)	0.10
End-stage	15 (9.9%)	4 (6.5%)	0.43
Liver disease			
Cirrhosis	7 (4.6%)	1 (1.6%)	0.44
Chronic			
Hepatitis B	0 (0.00%)	0 (0.00%)	>0.999
Hepatitis C	1 (0.66%)	0 (0.00%)	>0.999
Metabolic disease			
Obesity (BMI ≥ 30–40)	65 (42.8%)	27 (43.5%)	0.92
Morbid obesity (BMI ≥ 40)	27 (17.8%)	15 (24.2%)	0.28
BMI	33.5 (32.1–34.9)	34.7 (32.4–37.0)	0.36
Diabetes	70 (46.1%)	25 (40.3%)	0.44
Modified Charlson Comorbidity Index	2.5 (2.1–2.9)	1.8 (1.2–2.4)	0.07

*Continuous variables represented by mean (95% CI) with p-values represented by independent samples t-test or Mann-Whitney U test as appropriate; categorical variables represented by count and (%) of group with p-values represented by Pearson's chi-squared test or Fisher's exact test as appropriate. COPD, chronic obstructive pulmonary disease; HIV, human immunodeficiency virus; BMI, body mass index. Bold values indicate p ≤ 0.05*.

### ICU Variables

The Pao_2_/Fio_2_ ratios and mSOFA scores were evaluated at the start time of intubation (Table 2). Pao_2_/Fio_2_ values were similar between the DEX Group and No DEX Group at time of intubation (132.7 vs. 122.8 mmHg; *p* = 0.40). The mSOFA scores were similar between the DEX Group and No DEX Group at time of intubation (8.0 vs. 8.3; *p* = 0.55). The DEX group had a higher proportion use of sedative midazolam (77.0 vs. 50.0%; *p* < 0.001), lorazepam (65.1 vs. 21.0%; *p* < 0.001), and ketamine (30.9 vs. 9.7%; *p* = 0.001) (Table 2). However, the DEX group had a similar proportion of sedative propofol use (96.1 vs. 88.7%; *p* = 0.06) and similar proportion use of any gamma-aminobutyric acid (GABA) receptor ligand sedative (98.7 vs. 100%; *p* > 0.999) as compared to the No DEX group. Analgesic opioid use was similar between the DEX and No DEX group (94.1 vs. 91.9%; *p* = 0.55) (Table 2). Patients in the DEX group received DEX infusion on average for a duration of 7.5 days (95% CI 6.5–8.5).

**Table 2:** ICU variables.

**Variables**	**Dexmedetomidine** ***n* = 152**	**No Dexmedetomidine** ***n* = 62**	***p-value***
At time of intubation Pao_2_/Fio_2_	132.7 (120.8–144.6)	122.8 (101.5–144.1)	0.40
At time of intubation mSOFA	8.0 (7.6–8.5)	8.3 (7.4–9.2)	0.55
Sedative use	152 (100%)	62 (100%)	>0.999
GABA receptor ligand (any) use	150 (98.7%)	62 (100%)	>0.999
Propofol	146 (96.1%)	55 (88.7%)	0.06
Midazolam	117 (77.0%)	31 (50.0%)	**<0.001**
Lorazepam	99 (65.1%)	13 (21.0%)	**<0.001**
Ketamine	47 (30.9%)	6 (9.7%)	**0.001**
Opioid use	143 (94.1%)	57 (91.9%)	0.55
Corticosteroid (any) use	85 (55.9%)	29 (46.8%)	0.22
Methylprednisolone	31 (20.4%)	12 (19.4%)	0.86
Dexamethasone	29 (19.1%)	4 (6.5%)	**0.02**
Hydrocortisone	42 (27.6%)	14 (22.6%)	0.45
Prednisone	17 (11.2%)	5 (8.1%)	0.50
Remdesivir use	29 (19.1%)	4 (6.5%)	**0.02**
Hydroxychloroquine use	52 (34.2%)	21 (33.9%)	0.96
Antibiotic (any) use	139 (91.4%)	50 (80.6%)	**0.03**
Azithromycin	46 (30.3%)	23 (37.1%)	0.33
Anticoagulant (any) use	143 (94.1%)	55 (88.7%)	0.25
Enoxaparin	128 (84.2%)	35 (56.5%)	**<0.001**
Bivalirudin	32 (21.1%)	6 (9.7%)	**0.05**
Heparin	88 (57.9%)	34 (54.8%)	0.68
Apixaban	39 (25.7%)	4 (6.5%)	**0.001**
Argatroban	7 (4.6%)	1 (1.6%)	0.44
Rivaroxaban	11 (7.2%)	2 (3.2%)	0.36
Warfarin	5 (3.3%)	3 (4.8%)	0.69
Fondaparinux	1 (0.66%)	0 (0.00%)	>0.999
Inhaled nitric oxide use	6 (3.9%)	2 (3.2%)	>0.999
Vasopressor use	142 (93.4%)	45 (72.6%)	**<0.001**
Paralytic / neuromuscular blockade use	106 (69.7%)	42 (67.7%)	0.77
Prone positioning use	99 (65.1%)	24 (38.7%)	**<0.001**
Renal replacement therapy use	3 (2.0%)	1 (1.6%)	>0.999
Extracorporeal membrane oxygenation use	5 (3.3%)	0 (0.00%)	0.32

*Continuous variables represented by mean (95% CI) with p-values represented by independent samples t-test or Mann-Whitney U test as appropriate; categorical variables represented by count and (%) of group with p-values represented by Pearson's chi-squared test or Fisher's exact test as appropriate. GABA, gamma-aminobutyric acid; Pao_2_/Fio_2_, ratio of the partial pressure of arterial oxygen to the fraction of inspired oxygen; mSOFA, modified sequential organ failure assessment. Bold values indicate p ≤ 0.05*.

The proportion of any corticosteroid use was similar between the DEX and No DEX Group (55.9 vs. 46.8%; *p* = 0.22) (Table 2). With regard to specific corticosteroids, dexamethasone use was higher in the DEX group (19.1 vs. 6.5%; *p* = 0.02). However, the DEX group as compared to the No DEX Group had a similar proportion use of methylprednisolone (20.4 vs. 19.4%; *p* = 0.86), hydrocortisone (27.6 vs. 22.6%; *p* = 0.45), and prednisone (11.2 vs. 8.1%; *p* = 0.50) (Table 2).

The DEX group had a higher proportion of patients receiving remdesivir (19.1 vs. 6.5%; *p* = 0.02), any antibiotic (91.4 vs. 80.6%; *p* = 0.03), any vasopressor (93.4 vs. 72.6%; *p* < 0.001), and prone positioning (65.1 vs. 38.7%; *p* < 0.001) (Table 2). The use of antibiotic azithromycin, which has been tested as a COVID-19 therapeutic, was similar between groups (30.3% DEX vs. 37.1% No DEX; *p* = 0.33). Between DEX vs. No DEX groups, there was similar use of hydroxychloroquine (34.2 vs. 33.9%; *p* = 0.96), any anticoagulant (94.1 vs. 88.7%; *p* = 0.25), inhaled nitric oxide (3.9 vs. 3.2%; *p* > 0.999), paralytic medication (69.7 vs. 67.7%; *p* = 0.77), renal replacement therapy (2.0 vs. 1.6%; *p* > 0.999), and extracorporeal membrane oxygenation (3.3 vs. 0%; *p* = 0.32) (Table 2). Standard protocols at RUSH hospitals included the ABCDEF bundle for optimizing patient recovery and outcomes (42, 43) and use of sequential compression device boots for deep vein thrombosis prophylaxis.

### Mortality Outcomes

From start time of intubation, 28-day mortality in the cohort receiving DEX was 27.0% as compared to 64.5% in the cohort that did not receive DEX (relative risk reduction 58.2%; 95% CI 42.4–69.6). The use of DEX was associated with reduced 28-day mortality on multivariable Cox regression analysis from time of intubation (aHR 0.19; 95% CI, 0.10–0.33; *p* < 0.001). The use of DEX was also associated with reduced 28-day mortality on unadjusted univariate Cox regression analysis from time of intubation (HR 0.25; 95% CI 0.16-0.39; *p* < 0.001).

**Figure 1:**
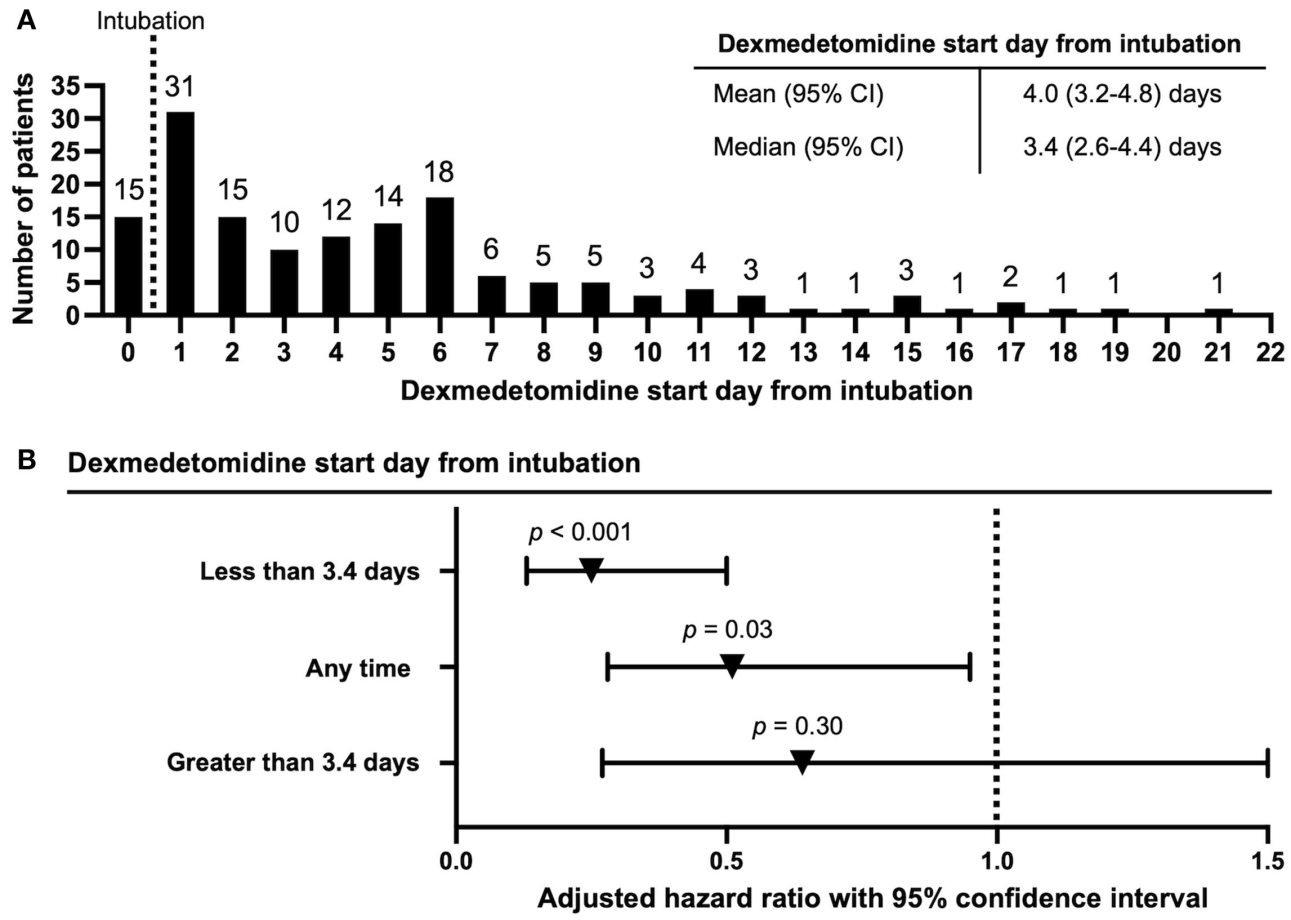
Dexmedetomidine start day from time of intubation and multivariable Cox regression assessing 28-day mortality from time of intubation accounting for time-varying exposure to dexmedetomidine. **(A)** Time to dexmedetomidine use from time of intubation. Day 0 includes patients already on dexmedetomidine prior to intubation. **(B)** Multivariable Cox regression assessing 28-day mortality from time of intubation accounting for dexmedetomidine as a time-varying covariate. Cut-off for dexmedetomidine start time from intubation included early start time (<3.4 days), later start time (>3.4 days), and all patients in the dexmedetomidine group (any time initiation of dexmedetomidine from intubation).

DEX was often started days post intubation (mean 4.0 days, median 3.4 days) (Figure 1A). Multivariable Cox regression assessing for 28-day mortality from time of intubation adjusting for time varying exposure to DEX revealed that DEX use was associated with reduced mortality (aHR 0.51; 95% CI 0.28–0.95; *p* = 0.03). Given the median start time of DEX at 3.4 days, we assessed DEX use prior to and after 3.4 days with DEX as a time varying covariate. There was a significant reduction in mortality in patients who received DEX prior to 3.4 days (aHR 0.25, 95% CI 0.13–0.50, *p* < 0.001). However, there was no significant associated reduction in mortality in patients that received DEX after 3.4 days from intubation (aHR 0.64, 95% CI 0.27–1.50, *p* = 0.30) (Figure 1B). As comparison, univariable Cox regression assessing for 28-day mortality from time of intubation adjusting for time varying exposure to DEX also demonstrated that DEX use was associated with reduced mortality (aHR 0.56; 95% CI 0.35–0.91; *p* = 0.02). In all primary adjusted and unadjusted analyses performed, DEX use was associated with reduced 28-day mortality from time of intubation (Table 3).

**Table 3:** 28-day mortality from time of intubation.

**Regression model**	**HR**	**aHR**	***p-value***
Multivariable^†^ Cox regression (DEX use)		0.19 (0.10–0.33)	**<0.001**
Univariable Cox Regression (DEX use)	0.25 (0.16–0.39)		**<0.001**
Multivariable^†^ Cox Regression (DEX use) with DEX use as a time-varying covariate		0.51 (0.28–0.95)	**0.03**
Univariable Cox Regression (DEX use) with DEX use as a time-varying covariate		0.56 (0.35–0.91)	**0.02**

*Values represented by hazard ratio (HR) or adjusted hazard ratio (aHR) (95% CI). Variables in multivariable analysis include: (i) DEX use (ii) age at hospital admission; (iii) body mass index (BMI) at hospital admission; (iv) mCCI at hospital admission; (v) Pao_2_/Fio_2_ at intubation; (vi) mSOFA at intubation; (vii) corticosteroid use; (viii) prone positioning use. DEX, dexmedetomidine. Bold values indicate p ≤ 0.05*.

Within multivariable Cox regression analyses, age and mSOFA score at time of intubation were associated with increased risk of mortality (Supplementary Tables 1–8). Any corticosteroid use was also associated with increased risk of mortality (Supplementary Tables 1, 2); however, in a separate analysis, dexamethasone use was associated with reduced mortality (Supplementary Tables 5, 6). Replacement of any corticosteroid use with dexamethasone in multivariable Cox regression demonstrated that DEX use was associated with reduced mortality when DEX was treated as a categorical variable (aHR 0.18; 95% CI 0.10–0.34; *p* < 0.001) or as a time-varying covariate when DEX was initiated at <3.4 days from intubation (aHR 0.26; 95% CI 0.13–0.52; *p* < 0.001) but not associated with reduced mortality when DEX was initiated > 3.4 days from intubation (aHR 0.69; 95% CI 0.29–1.64; *p* = 0.40) or when assessed at all time points when DEX was treated as a time varying covariate (aHR 0.57; 95% CI 0.31−1.06; *p* = 0.07) (Supplementary Tables 5–8).

## Discussion

We performed a retrospective analysis to assess mortality associated with α_2_ AR agonist use in patients with COVID-19. To achieve this objective, we evaluated use of DEX (an ICU sedative) in critically ill patients with COVID-19 requiring sedation and invasive mechanical ventilation. DEX use was associated with reduced 28-day mortality from time of intubation on all primary multivariable and univariable Cox regression analyses. Furthermore, initiation of DEX use <3.4 days from time of intubation was associated with reduced mortality, while later initiation of DEX was not associated with reduced mortality.

In multivariable Cox regression analyses, we adjusted for covariates that could have influenced mortality outcomes (age, BMI, mCCI, Pao_2_/Fio_2_, mSOFA, prone positioning, and corticosteroid use). Furthermore, we adjusted for time varying exposure to DEX and accounted for immortal time bias. Adjusting for confounding immortal time bias can significantly influence the association of the drug of interest with mortality as compared to unadjusted analyses (12).

Since initiation of our analysis, there have been recent reports on the use of α_2_ AR agonist use in COVID-19 outcomes. In one case series, early administration of α_2_ AR agonist clonidine appeared to mitigate progression of moderate to severe COVID-19, when provided before or at the time of requirement of oxygenation or hospitalization (44). Intriguingly, the authors chose clonidine for the dual purpose of its anti-hypertensive effects and immunomodulatory effects. A potential benefit of clonidine or even lower dose DEX use would be that these drugs at lower doses only mildly sedate patient (minimal effect on patient awareness) and could potentially be given to patients with COVID-19 not requiring oxygenation or hospitalization. Given that there is no benefit and potential harm in providing patients with immunosuppressive corticosteroids prior to oxygenation requirements in COVID-19 (9), an α_2_ AR agonist could potentially be used as an immunomodulator in the earlier stages of COVID-19—prior to requirement of oxygenation—where corticosteroids have been ineffective (9).

A recent retrospective study analyzed patients over 12 h following initiation of DEX administration; patients receiving DEX had improvement in oxygenation (Pao_2_/Fio_2_ ratio) over the 12 h assessment time period (45). Similarly, in a case report, a patient was found to have gradually worsening hypoxemia on non-invasive ventilation, and intubation was strongly considered; however the patient was trialed on DEX and subsequent of improvement oxygenation followed without need for intubation; the authors hypothesized this may be due to behavior changes (agitation to calm) or physiologic changes induced by the drug (46); outside of the potential immunomodulator benefits of DEX, it is relevant to consider DEX may be helping to improve oxygenation through other modalities such as reduced agitation and increased ventilator compliance as well as improvement in respiratory mechanics, enhanced hypoxic pulmonary vasoconstriction and improvement in ventilation / perfusion ratio (25, 26, 32–35).

Other recent studies in combination with ours suggest that α_2_ AR agonist use when administered around the time of or prior to hospitalization and oxygen requirement or around the time of invasive mechanical ventilation may provide outcome benefits for patients with COVID-19. We found patients receiving DEX closer to the time of intubation had improved associated mortality outcomes as compared to later DEX use. Once SARS-CoV-2 virus gains entry into host cells, it begins eliciting local inflammation. This can contribute to local organ damage and dysfunction. Local infection and inflammation can propagate, and a systemic hyperinflammatory response can result, causing further organ damage and dysfunction (4). After organ damage from sustained inflammation and immune cell response, injury can be irreversible. Optimal initiation of an α_2_ AR agonist may be before or at the time of invasive mechanical ventilation, in an attempt to prevent immune mediated organ dysfunction and irreversible organ damage.

Steroids such as dexamethasone, methylprednisolone, and hydrocortisone have been investigated in RCTs, and these trials overall have demonstrated improved mortality outcomes when steroids are used as an immunosuppressant for COVID-19 treatment (9, 10). However, within the majority of time of our analysis, a standard protocol for corticosteroid use in COVID-19 was not in place. During the majority of the study period (March 1, 2020 to July 30, 2020), corticosteroid use initiated at the time of oxygenation or invasive mechanical ventilation was controversial due to concerns of suppressed viral clearance, and use of corticosteroids was variable for COVID-19 patients. Corticosteroids are also employed for the management of hypotension and vasopressor dependent shock in the critical care setting (47), which would be associated with increased risk of mortality. The associated increased risk of mortality with any corticosteroid use in our study could be reflective of management of more severe course of COVID-19. However, starting in July 2020, strong evidence demonstrated corticosteroid use (dexamethasone) initiated at the time of requirement of oxygen support or invasive mechanical ventilation improved mortality outcomes (9), and patients at RUSH hospitals began being treated based on these guidelines. In this study, 19.1% of patients in the DEX group received dexamethasone as compared to 6.5% of patients in the No DEX group (*p* = 0.02). Dexamethasone use was associated with reduced mortality and did influence the association of mortality benefit of DEX in some of our multivariable analyses. Our results demonstrated DEX use within 3.4 days from time of intubation is associated with reduced mortality when dexamethasone use was specifically incorporated in the multivariable Cox regression model. However, studies performed after July 2020 would be able to assess a greater number of patients receiving continuous corticosteroids initiated between the time of initiation of oxygen support and invasive mechanical ventilation for COVID-19.

While no results have been posted to date, current clinical trials (ClinicalTrials.Gov) may provide further insight into use of DEX initiation from the start of non-invasive ventilation (NCT04358627) or in patients that have been intubated (NCT04413864) on COVID-19 outcomes and inflammation. These results and additional studies should be of high importance to evaluate α_2_ AR agonists and their potential to limit COVID-19 disease severity and potentially mortality. Furthermore, as discussed in the introduction, since α_2_ AR agonists may share overlap in function as an immunomodulator as compared to α_1_ AR antagonists, insights from the clinical trial investigating an α_1_ AR antagonist in the potential to reduce COVID-19 mortality (NCT04365257) may be applicable.

Our current study is limited in being retrospective, which prevents standardization of treatment between groups. Our assessment was restricted to a single hospital system with a limited number of patients (*n* = 214). Use of DEX varies between hospital systems, and our current analysis was restricted to RUSH hospitals. Further retrospective studies expanded to other hospital systems as well as results from RCTs are needed to evaluate DEX and potential to reduce COVID-19 mortality. A strength of this analysis includes adjustment for confounders not only with multivariable Cox regression but also with evaluation of the drug of interest (DEX) as a time-varying covariate (12); this has been described as an important assessment in COVID-19 mortality outcomes that is often not implemented (48). Regardless, more study results are urgently needed to evaluate the potential impact of α_2_ AR agonists on COVID-19 mortality as currently addressed.

In summary, use of α_2_ AR agonist DEX was associated with lower mortality in critically ill patients with COVID-19 requiring invasive mechanical ventilation at RUSH hospitals on retrospective analysis. The associated mortality benefit of DEX appeared to be related to earlier use closer to the time of intubation as opposed to later use. The use of an α_2_ AR agonist might be an important pharmacologic agent in patients with COVID-19 to reduce mortality. While limited studies, including ours, report benefits using α_2_ AR agonists, such as DEX and clonidine, in COVID-19 outcomes, larger retrospective analyses expanded to other hospital systems and RCTs are needed to further explore these findings.

## Data Availability Statement

The original contributions presented in the study are included in the article/Supplementary Material, further inquiries can be directed to the corresponding authors.

## Ethics Statement

The studies involving human participants were reviewed and approved by Institutional Review Board at Rush University Medical Center. Written informed consent for participation was not required for this study in accordance with the national legislation and the institutional requirements.

## Author Contributions

JH, MV, LF, MW, and RB developed the study design. JH, MV, EK, LF, MW, and RB conducted statistical analysis. JH, MW, and RB wrote the manuscript with input from all authors. The final manuscript was reviewed by all authors.

## Funding

NIH grant 1T32AR073157-01A1 (to JH and MW); Grainger Chair of the Rush Arthritis and Orthopedics Institute (to MW); Rush Bioinformatics and Biostatistics Core with support from NIH grant 5UL1TR002389-04 (Julian Solway).

## Conflict of Interest

JH and MW have filed a non-provisional patent application pertaining to work associated with this study. The remaining authors declare that the research was conducted in the absence of any commercial or financial relationships that could be construed as a potential conflict of interest.

## Publisher's Note

All claims expressed in this article are solely those of the authors and do not necessarily represent those of their affiliated organizations, or those of the publisher, the editors and the reviewers. Any product that may be evaluated in this article, or claim that may be made by its manufacturer, is not guaranteed or endorsed by the publisher.
