# Supplementary material for: The Association of an Alpha-2 Adrenergic Receptor Agonist and Mortality in Patients With COVID-19
Source: Front Med (Lausanne). 2022 Jan 4;8:797647. doi: 10.3389/fmed.2021.797647 (PMC8764306; doi:10.3389/fmed.2021.797647)
Supplement: Supplementary file 1 [file Data_Sheet_1.docx]

**The Association of an Alpha-2 Adrenergic Receptor Agonist and Mortality in Patients with COVID-19**

John L. Hamilton M.D, Ph.D., Mona Vashi M.D., Ekta B. Kishen M.P.H., Louis F. Fogg Ph.D., Markus A. Wimmer Ph.D., Robert A. Balk M.D.

**Supplementary Appendix**

**Table of Contents**

**Table S1: Multivariable Cox regression 28-day mortality from intubation** 2

- **Covariates:** Age, BMI, modified Charlson Comorbidity Index, Pao_2_/FIo_2_ at intubation, modified SOFA at intubation, corticosteroid use (any), prone positioning, dexmedetomidine use

**Table S2: Multivariable Cox regression 28-day mortality from intubation** 3

- **Dexmedetomidine as a time varying covariate**
- **Covariates:** Age, BMI, modified Charlson Comorbidity Index, Pao_2_/FIo_2_ at intubation, modified SOFA at intubation, corticosteroid use (any), prone positioning, dexmedetomidine as a time varying covariate

**Table S3: Multivariable Cox regression 28-day mortality from intubation** 4

- **Dexmedetomidine as a time varying covariate**
- **Dexmedetomidine start time < 3.4 days from intubation**
- **Covariates:** Age, BMI, modified Charlson Comorbidity Index, Pao_2_/FIo_2_ at intubation, modified SOFA at intubation, corticosteroid use (any), prone positioning, dexmedetomidine as a time varying covariate

**Table S4: Multivariable Cox regression 28-day mortality from intubation** 4

- **Dexmedetomidine as a time varying covariate**
- **Dexmedetomidine start time > 3.4 days from intubation**
- **Covariates:** Age, BMI, modified Charlson Comorbidity Index, Pao_2_/FIo_2_ at intubation, modified SOFA at intubation, corticosteroid use (any), prone positioning, dexmedetomidine as a time varying covariate

**Table S5: Multivariable Cox regression 28-day mortality from intubation** 5

- **Covariate corticosteroid use (any) replaced with dexamethasone use**
- **Covariates:** Age, BMI, modified Charlson Comorbidity Index, Pao_2_/FIo_2_ at intubation, modified SOFA at intubation, dexamethasone use, prone positioning, dexmedetomidine use

**Table S6: Multivariable Cox regression 28-day mortality from intubation** 6

- **Dexmedetomidine as a time varying covariate**
- **Covariate corticosteroid use (any) replaced with dexamethasone use**
- **Covariates:** Age, BMI, modified Charlson Comorbidity Index, Pao_2_/FIo_2_ at intubation, modified SOFA at intubation, dexamethasone use, prone positioning, dexmedetomidine as a time varying covariate

**Table S7: Multivariable Cox regression 28-day mortality from intubation** 7

- **Dexmedetomidine as a time varying covariate**
- **Dexmedetomidine start time < 3.4 days from intubation**
- **Covariate corticosteroid use (any) replaced with dexamethasone use**
- **Covariates:** Age, BMI, modified Charlson Comorbidity Index, Pao_2_/FIo_2_ at intubation, modified SOFA at intubation, dexamethasone use, prone positioning, dexmedetomidine as a time varying covariate

**Table S8: Multivariable Cox regression 28-day mortality from intubation** 7

- **Dexmedetomidine as a time varying covariate**
- **Dexmedetomidine start time > 3.4 days from intubation**
- **Covariate corticosteroid use (any) replaced with dexamethasone use**
- **Covariates:** Age, BMI, modified Charlson Comorbidity Index, Pao_2_/FIo_2_ at intubation, modified SOFA at intubation, dexamethasone use, prone positioning, dexmedetomidine as a time varying covariate

| **Table S1: Multivariable Cox regression 28-day mortality from intubation**  **Covariates:** Age, BMI, modified Charlson Comorbidity Index, Pao_2_/FIo_2_ at intubation, modified SOFA at intubation, corticosteroid use (any), prone positioning, dexmedetomidine use | | | |
| --- | --- | --- | --- |
| **Variables** | **aHR** | **95% CI** | ***p*** |
| Age | 1.04 | 1.02-1.07 | **<0.001** |
| Body mass index | 1.00 | 0.96-1.03 | 0.87 |
| Modified Charlson Comorbidity Index | 1.04 | 0.91-1.19 | 0.58 |
| Pao_2_/FIo_2_ at intubation | 1.00 | 1.00-1.01 | 0.82 |
| Modified SOFA at intubation | 1.40 | 1.21-1.62 | **<0.001** |
| Corticosteroid (any) use | 2.51 | 1.25-5.07 | **0.01** |
| Prone positioning | 1.08 | 0.53-2.21 | 0.83 |
| Dexmedetomidine use | 0.19 | 0.10-0.33 | **<0.001** |
| aHR: adjusted hazard ratio; 95% CI: 95% confidence interval | | | |

| **Table S2: Multivariable Cox regression 28-day mortality from intubation**  **Dexmedetomidine as a time varying covariate**  **Covariates:** Age, BMI, modified Charlson Comorbidity Index, Pao_2_/FIo_2_ at intubation, modified SOFA at intubation, corticosteroid use (any), prone positioning, dexmedetomidine as a time varying covariate | | | |
| --- | --- | --- | --- |
| **Variables** | **aHR** | **95% CI** | ***p*** |
| Age | 1.04 | 1.02-1.07 | **0.001** |
| Body mass index | 1.00 | 0.96-1.03 | 0.92 |
| Modified Charlson Comorbidity Index | 0.98 | 0.86-1.11 | 0.71 |
| Pao_2_/FIo_2_ at intubation | 1.00 | 0.99-1.00 | 0.91 |
| Modified SOFA at intubation | 1.40 | 1.22-1.61 | **<0.001** |
| Corticosteroid use | 2.51 | 1.29-4.90 | **0.007** |
| Prone positioning | 0.81 | 0.41-1.57 | 0.53 |
| Dexmedetomidine (time varying exposure) | 0.51 | 0.28-0.95 | **0.03** |
| aHR: adjusted hazard ratio; 95% CI: 95% confidence interval | | | |

| **Table S3: Multivariable Cox regression 28-day mortality from intubation**  **Dexmedetomidine as a time varying covariate**  **Dexmedetomidine start time < 3.4 days from intubation**  **Covariates:** Age, BMI, modified Charlson Comorbidity Index, Pao_2_/FIo_2_ at intubation, modified SOFA at intubation, corticosteroid use (any), prone positioning, dexmedetomidine as a time varying covariate | | | |
| --- | --- | --- | --- |
| **Variables** | **aHR** | **95% CI** | ***p*** |
| Age | 1.04 | 1.01-1.07 | **0.004** |
| Body mass index | 1.01 | 0.97-1.05 | 0.69 |
| Modified Charlson Comorbidity Index | 1.01 | 0.86-1.20 | 0.86 |
| Pao_2_/FIo_2_ at intubation | 1.00 | 0.99-1.01 | 0.93 |
| Modified SOFA at intubation | 1.43 | 1.22-1.69 | **<0.001** |
| Corticosteroid use | 1.57 | 0.73-3.39 | 0.25 |
| Prone positioning | 1.33 | 0.60-2.97 | 0.49 |
| Dexmedetomidine (time varying exposure) | 0.25 | 0.13-0.50 | **<0.001** |
| aHR: adjusted hazard ratio; 95% CI: 95% confidence interval | | | |

| **Table S4: Multivariable Cox regression 28-day mortality from intubation**  **Dexmedetomidine as a time varying covariate**  **Dexmedetomidine start time > 3.4 days from intubation**  **Covariates:** Age, BMI, modified Charlson Comorbidity Index, Pao_2_/FIo_2_ at intubation, modified SOFA at intubation, corticosteroid use (any), prone positioning, dexmedetomidine as a time varying covariate | | | |
| --- | --- | --- | --- |
| **Variables** | **aHR** | **95% CI** | ***p*** |
| Age | 1.06 | 1.03-1.10 | **<0.001** |
| Body mass index | 1.01 | 0.97-1.05 | 0.68 |
| Modified Charlson Comorbidity Index | 0.93 | 0.78-1.11 | 0.40 |
| Pao_2_/FIo_2_ at intubation | 1.00 | 0.99-1.00 | 0.66 |
| Modified SOFA at intubation | 1.40 | 1.15-1.70 | **0.001** |
| Corticosteroid use | 2.65 | 1.10-6.39 | **0.03** |
| Prone positioning | 0.40 | 0.15-1.03 | 0.06 |
| Dexmedetomidine (time varying exposure) | 0.64 | 0.27-1.50 | 0.30 |
| aHR: adjusted hazard ratio; 95% CI: 95% confidence interval | | | |

| **Table S5: Multivariable Cox regression 28-day mortality from intubation**  **Covariate corticosteroid use (any) replaced with dexamethasone use**  **Covariates:** Age, BMI, modified Charlson Comorbidity Index, Pao_2_/FIo_2_ at intubation, modified SOFA at intubation, dexamethasone use, prone positioning, dexmedetomidine use | | | |
| --- | --- | --- | --- |
| **Variables** | **aHR** | **95% CI** | ***p*** |
| Age | 1.04 | 1.02-1.06 | **0.001** |
| Body mass index | 0.99 | 0.95-1.03 | 0.63 |
| Modified Charlson Comorbidity Index | 1.06 | 0.91-1.2 | 0.44 |
| Pao_2_/FIo_2_ at intubation | 1.00 | 0.99-1.00 | 0.82 |
| Modified SOFA at intubation | 1.36 | 1.17-1.58 | **<0.001** |
| Dexamethasone use | 0.30 | 0.09-0.97 | **0.04** |
| Prone positioning | 1.81 | 0.92-3.55 | 0.09 |
| Dexmedetomidine use | 0.18 | 0.10-0.34 | **<0.001** |
| aHR: adjusted hazard ratio; 95% CI: 95% confidence interval | | | |

| **Table S6: Multivariable Cox regression 28-day mortality from intubation**  **Dexmedetomidine as a time varying covariate**  **Covariate corticosteroid use (any) replaced with dexamethasone use**  **Covariates:** Age, BMI, modified Charlson Comorbidity Index, Pao_2_/FIo_2_ at intubation, modified SOFA at intubation, dexamethasone use, prone positioning, dexmedetomidine as a time varying covariate | | | |
| --- | --- | --- | --- |
| **Variables** | **aHR** | **95% CI** | ***p*** |
| Age | 1.04 | 1.01-1.06 | **0.002** |
| Body mass index | 0.99 | 0.96-1.03 | 0.76 |
| Modified Charlson Comorbidity Index | 0.97 | 0.85-1.11 | 0.68 |
| Pao_2_/FIo_2_ at intubation | 1.00 | 0.99-1.00 | 0.66 |
| Modified SOFA at intubation | 1.36 | 1.18-1.56 | **<0.001** |
| Dexamethasone use | 0.28 | 0.09-0.91 | **0.03** |
| Prone positioning | 1.15 | 0.61-2.17 | 0.66 |
| Dexmedetomidine (time varying exposure) | 0.57 | 0.31-1.06 | 0.07 |
| aHR: adjusted hazard ratio; 95% CI: 95% confidence interval | | | |

| **Table S7: Multivariable Cox regression 28-day mortality from intubation**  **Dexmedetomidine as a time varying covariate**  **Dexmedetomidine start time < 3.4 days from intubation**  **Covariate corticosteroid use (any) replaced with dexamethasone use**  **Covariates:** Age, BMI, modified Charlson Comorbidity Index, Pao_2_/FIo_2_ at intubation, modified SOFA at intubation, dexamethasone use, prone positioning, dexmedetomidine as a time varying covariate | | | |
| --- | --- | --- | --- |
| **Variables** | **aHR** | **95% CI** | ***p*** |
| Age | 1.04 | 1.01-1.06 | **0.006** |
| Body mass index | 1.01 | 0.97-1.05 | 0.63 |
| Modified Charlson Comorbidity Index | 1.04 | 0.87-1.24 | 0.68 |
| Pao_2_/FIo_2_ at intubation | 1.00 | 0.99-1.00 | 0.88 |
| Modified SOFA at intubation | 1.41 | 1.20-1.66 | **<0.001** |
| Dexamethasone use | 0.38 | 0.11-1.29 | 0.12 |
| Prone positioning | 1.91 | 0.88-4.13 | 0.10 |
| Dexmedetomidine (time varying exposure) | 0.26 | 0.13-0.52 | **<0.001** |
| aHR: adjusted hazard ratio; 95% CI: 95% confidence interval | | | |

| **Table S8: Multivariable Cox regression 28-day mortality from intubation**  **Dexmedetomidine as a time varying covariate**  **Dexmedetomidine start time > 3.4 days from intubation**  **Covariate corticosteroid use (any) replaced with dexamethasone use**  **Covariates:** Age, BMI, modified Charlson Comorbidity Index, Pao_2_/FIo_2_ at intubation, modified SOFA at intubation, dexamethasone use, prone positioning, dexmedetomidine as a time varying covariate | | | |
| --- | --- | --- | --- |
| **Variables** | **aHR** | **95% CI** | ***p*** |
| Age | 1.05 | 1.02-1.08 | **0.001** |
| Body mass index | 1.01 | 0.96-1.05 | 0.95 |
| Modified Charlson Comorbidity Index | 0.96 | 0.81-1.14 | 0.63 |
| Pao_2_/FIo_2_ at intubation | 1.00 | 0.99-1.00 | 0.89 |
| Modified SOFA at intubation | 1.34 | 1.10-1.64 | **0.004** |
| Dexamethasone use | 0.21 | 0.03-1.57 | 0.13 |
| Prone positioning | 0.74 | 0.32-1.71 | 0.49 |
| Dexmedetomidine (time varying exposure) | 0.69 | 0.29-1.64 | 0.40 |
| aHR: adjusted hazard ratio; 95% CI: 95% confidence interval | | | |
